# PER, a Circadian Clock Component, Mediates the Suppression of MMP-1 Expression in HaCaT Keratinocytes by cAMP

**DOI:** 10.3390/molecules23040745

**Published:** 2018-03-23

**Authors:** Miji Yeom, HansongI Lee, Seoungwoo Shin, Deokhoon Park, Eunsun Jung

**Affiliations:** Biospectrum Life Science Institute, A-1805, U-TOWER, 767, Sinsu-ro, Suji-gu 16827, Yongin-si, Gyeonggi-do, Korea; biout@biospectrum.com (M.Y.); bioyw@biospectrum.com (H.L.); biost@biospectrum.com (S.S.); pdh@biospectrum.com (D.P.)

**Keywords:** skin circadian clock, PER, MMP-1, cAMP, *Lespedeza capitata*

## Abstract

Skin circadian clock system responds to daily changes, thereby regulating skin functions. Exposure of the skin to UV irradiation induces the expression of matrix metalloproteinase-1 (MMP-1) and causes DNA damage. It has been reported both DNA repair and DNA replication are regulated by the circadian clock in mouse skin. However, the molecular link between circadian clock and MMP-1 has little been investigated. We found PERIOD protein, a morning clock component, represses the expression of MMP-1 in human keratinocytes by using a PER-knockdown strategy. Treatment with siPer3 alleviated the suppression of MMP-1 expression induced by forskolin. Results revealed PER3 suppresses the expression of MMP-1 via cAMP signaling pathway. Additionally, we screened for an activator of PER that could repress the expression of MMP-1 using HaCaT cell line containing *PER* promoter-luciferase reporter gene. Results showed *Lespedeza capitate* extract (LCE) increased *PER* promoter activity. LCE inhibited the expression of MMP-1 and its effect of LCE was abolished in knockdown of PER2 or PER3, demonstrating LCE can repress the expression of MMP-1 through PER. Since circadian clock component PER can regulate MMP-1 expression, it might be a new molecular mechanism to develop therapeutics to alleviate skin aging and skin cancer.

## 1. Introduction

The circadian clock is an endogenous timing mechanism that allows anticipation of regular daily changes and operation of biological processes at proper time during a day. Studies in mice have shown that a disruption of internal rhythms causes premature aging and reduces life span and fertility rates, thereby having negative consequences for fitness [1,2]. In mammals, the central clock is in the suprachiasmatic nucleus (SCN) of the hypothalamus, which receives environmental timing 

Information through the retina. It is essential for synchronization of organisms with periodic environmental changes [3,4]. Most peripheral tissues have self-sustaining oscillators synchronized by the central clock via systemic time cues such as hormonal and neuronal signals [5,6]. Peripheral clocks regulate local physiology in a circadian manner [7]. At a molecular level, the circadian clock consists of interlocked transcription-translation feedback loop of PER 1–3 (Period 1–3) and Cry 1–2 (Crytochromes 1 and 2) genes. Expression of these genes is activated by transcription factors BMAL1 (brain and muscle aryl hydrocarbon receptor nuclear translocator (ARNT)-like protein-1) and CLOCK (circadian locomotor output cycles kaput) [8].

Since skin is positioned at the interface between body and environment, it is subjected to daily variations in environments such as light, temperature, UV radiation, humidity, and pathogens. Many human skin properties oscillate in a circadian manner including cell proliferation rates, hydration and transepidermal water loss (TEWL), sebum production, surface pH, temperature, and facial rhytides [9,10,11,12,13,14]. It has been reported that clock genes *BMAL1*, *CLOCK*, *PER*, and *CRY* are expressed in cultured human skin cells including keratinocytes, melanocytes, and dermal fibroblasts [15]. Each of these diverse skin cell types contains distinct circadian clock machinery that autonomously drives daily functions. At molecular level, core clock component can regulate approximately 2–10% of total genome in each tissue including skin [16,17]. Disruption of core circadian clock genes affects the gene expression and cellular processes important for skin function.

Acute and chronic exposure of the skin to UV irradiation induces MMP-1 expression and causes DNA damage, ultimately leading to premature skin aging (photoaging) and skin cancer [18,19]. It has been reported that both DNA repair, which is a mechanism for removing UV photoproducts, and DNA replication are regulated by the circadian clock in mouse skin. Oscillation of xeroderma pigmentosum complementation group A (XPA), one of six core factors important for removing UV photoproducts from DNA, has the lowest level during the night, in parallel with oscillation of repair activity [20,21,22]. It has been reported that the highest proportion of epidermal progenitor/stem cell proliferation in mice is in S-phase during the night [9]. 

MMPs is composed of a family of zinc-containing proteinases that are responsible for degradation of extracellular matrix (ECM) proteins such as collagen, fibronectin, elastin, and proteoglycans, contributing to photoaging [23,24,25]. The major enzyme responsible for collagen 1 digestion, MMP-1, is induced by exposure to sunlight [26]. UVB-irradiated keratinocytes stimulate MMP-1 release from fibroblasts. Also, damaged keratinocytes activate MMP-1 release by self. MMP-1 secreted from keratinocytes and fibroblasts impair the structural integrity of the dermis and result in the re-modelling of the skin [27,28]. MMP-1 expression is regulated by diverse signaling pathways. Of those pathways, cAMP signaling can inhibit MMP-1 expression in human skin fibroblasts [29]. cAMP signaling pathways also has been involved in circadian clock entraining and light-induced clock resetting [30]. Activated PKA by cAMP stimulates cAMP-response element binding protein (CREB) which induces cellular genes containing cAMP-response element (CRE) including *PER*. Light activation of *PER* genes is achieved through CREB/CRE signaling [31,32,33]. Although there are clues about the relation between MMP-1 and circadian clock, a direct molecular link between the circadian clock and MMP-1 has little been investigated.

To understand the molecular link between the circadian clock and MMP-1, we examined the effect of circadian clock on MMP-1 expression in human adult low calcium temperature (HaCaT) keratinocytes after knocking down core clock genes. We found that knockdown of PER increased the expression of MMP-1 regulated by cAMP signaling. Furthermore, we found that *Lespedeza capitata* extract could inhibit the expression of MMP-1 by activating the expression of *PER* gene. Our results suggest that the circadian clock protein PER might play a key role in regulating MMP-1 expression.

## 2. Results

### 2.1. Knockdown of PER Proteins Increase the Expression of MMP-1 in HaCaT Cells

**Figure 1:**
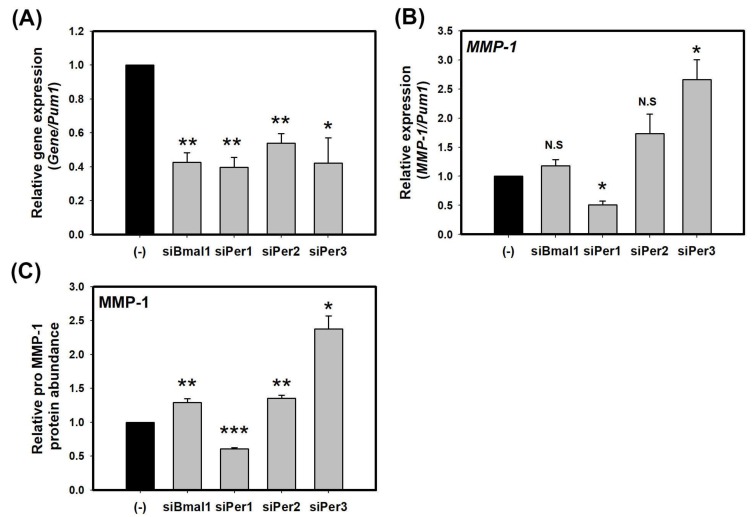
Core clock proteins regulate the expression of MMP-1 in HaCaT cells. (**A**) Transcriptional levels of endogenous clock genes in HaCaT cells transfected with control-siRNA, siBmal1, siPer1, siPer2, and siPer3 for 72 h; (**B**) Levels of *MMP-1* transcripts in HaCaT cells transfected with control-siRNA, siBmal1, siPer1, siPer2, and siPer3 for 72 h; (**C**) Levels of pro MMP-1 proteins in cell supernatants of HaCaT cells transfected with control-siRNA, siBmal1, siPer1, siPer2, and siPer3 for 72 h. Data are presented as mean ± SEM from three replicated measurements. Data is the relative value compared with control group. (Two-tail unpaired *t*-test, *n* = 3, * *p* < 0.05, ** *p* < 0.01, *** *p* < 0.001 compared with control-siRNA, N.S.: not significant).

To study the potential impact of the circadian clock on MMP-1, we investigated whether core clock protein, BMAL1, PER1, PER2, and PER3 could directly regulate the expression of MMP-1. HaCaT cells were transfected with each of *BMAL1-*, *PER1-*, *PER2-,* and *PER3*-siRNA. At 72 h after transfection, total RNA was extracted from transfected cells. Endogenous levels of *BMAL1*, *PER1*, *PER2*, and *PER3* were effectively knocked down by their respective siRNA (about 60% knockdown by *BMAL1-*, *PER1-,* and *PER3-*siRNA and about 50% knockdown by *PER2*-siRNA compared to a scrambled nontargeting siRNA) based on quantitative real-time PCR results (Figure 1A). Cells transfected with each of *BMAL1-*, *PER1-*, *PER2-,* and *PER3*-siRNA were used to measure transcriptional levels of *MMP-1*. Knockdown of PER2 and PER3 increased transcriptional levels of *MMP-1* as much as two or three times while knockdown of BMAL1 slightly increased transcriptional levels of *MMP-1* compared to non-knockdown control treated with control-siRNA. However, knockdown of PER1 decreased transcriptional levels of *MMP-1* by about half compared to control-siRNA treatment (Figure 1B). Secreted MMP-1 protein levels were determined by ELISA. The results revealed that treatment with *BMAL1-*, *PER2-*, and *PER3*-siRNA increased secreted MMP-1 protein levels (Figure 1C), similar to results obtained by real-time PCR. Among different treatments, knockdown of PER3 showed the strongest effect on the expression of *MMP-1*. These results revealed that core clock proteins could directly regulate the expression of *MMP-1*. Especially, PER2 and PER3, as components of morning clock could function as inhibitors of *MMP-1* expression. 

### 2.2. Suppression of MMP-1 by cAMP is Mediated by PER

**Figure 2:**
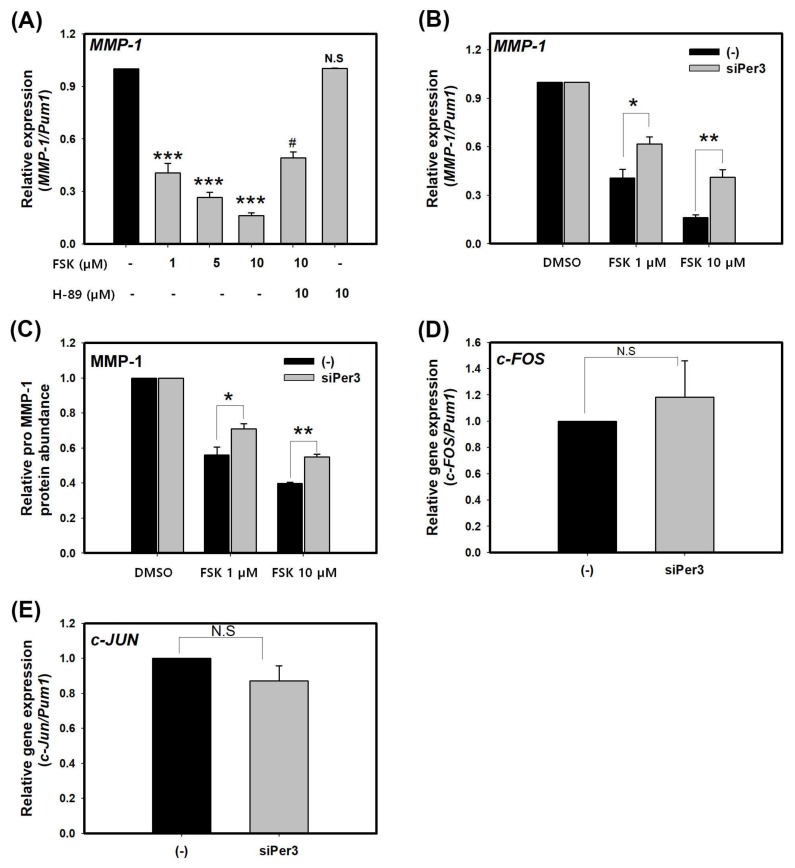
cAMP suppresses the expression of MMP-1 through PER3 in HaCaT cells. (**A**) Levels of MMP-1 transcripts in HaCaT cells treated with DMSO (-), FSK, and H-89 at indicated concentration for 24 h (two-tail unpaired, *n* = 3, * *p* < 0.05, ** *p* < 0.01, *** *p* < 0.001, N.S.: not significant compared with DMSO only (-) and ^#^ *p* < 0.05 compared with 10 uM FSK); (**B**) Levels of *MMP-1* transcripts in HaCaT cells treated with control-siRNA or siPer3 for 48 h, followed by co-treatment with FSK and siRNA for 24 h; (**C**) Levels of pro MMP-1 proteins in cell supernatants of HaCaT treated with control-siRNA or siPer3 for 48 h, followed by co-treatment with FSK and siRNA for 24 h (in **B**,**C**, one-way ANOVA, followed by Bonferroni post hoc, *n* = 3, * *p* < 0.05, ** *p* < 0.01 compared with control-siRNA at the indicated concentration) levels of c-Fos (**D**) and c-Jun (**E**) transcripts in aCaT cells treated with control-siRNA or siPer3 for 72 h (Two-tail unpaired, *n* = 3, N.S.: not significant compared with control-siRNA). Data are presented as mean ± SEM from three replicated measurements. Data is the relative value compared with control group in (**A**,**D**,**E**) or compared with respective control group (control-siRNA or siPer3 at DMSO) in (**B**,**C**).

Expression of MMP-1 can be activated by diverse external and internal stimuli such as UV radiation, toxic exposure, oxidative stress, and aging. It is likely to be regulated by many signaling pathways. Of those pathways, cAMP signaling can inhibit MMP-1 expression in human skin fibroblasts [29]. cAMP-dependent signaling plays an important role in sustaining circadian oscillation through cAMP-response element (CRE) [31,32,33]. Here, we investigated the involvement of cAMP signaling in the inhibitory effect of PER on expression of MMP-1 in keratinocytes. To examine whether an increase of cAMP could influence the expression of *MMP-1*, HaCaT cells were treated with forskolin (FSK), a cAMP-inducing agent, at indicated concentrations. FSK suppressed transcriptional levels of *MMP-1* in a dose-dependent manner. After treatment with 10 μM FSK, transcriptional levels of *MMP-1* was significantly decreased (by about 80%) (Figure 2A). It has been reported that many biological processes of cAMP signaling are mediated through PKA pathway [34,35,36]. To examine whether PKA pathway is involved in the suppression of *MMP-1* by cAMP, we treated HaCaT cells with both H-89 (a specific a PKA inhibitor, 10 μM) and FSK. Transcriptional level of *MMP-1* from cells co-treated with H-89 and FSK was increased about three times when compared to that from cells treated with FSK only (Figure 2A). These results showed that H-89 could antagonize the suppression of *MMP-1* expression by cAMP, indicating that PKA activity might mediate the effect of cAMP on *MMP-1* expression. 

To determine whether PER could suppress the expression of MMP-1 through cAMP signaling, we tested the effect of FSK on MMP-1 expression after knockdown of PER3 because siPer3 showed the strongest effect on the expression of MMP-1. We tested the effect of PER3 on expression of MMP-1 through cAMP signaling. In the control-siRNA group, the transcriptional levels of *MMP-1* was significantly decreased by treatment with FSK in a dose-dependent manner. However, in the siPer3 treatment group, the inhibitory effect of FSK on *MMP-1* mRNA was significantly attenuated compared to that in the control siRNA group (Figure 2B). With respect to the secreted MMP-1 protein levels, cells transfected with control siRNA showed significant decrease compared to cells transfected with siPer3 (Figure 2C), similar to real-time PCR results at mRNA level. These results suggest that cAMP signaling contributes to the suppression of MMP-1 and this signaling is partially mediated by clock protein PER3. 

A previous report has shown that cAMP signaling represses MMP-1 expression through PKA-mediated inhibition of extracellular signal-regulated kinase (ERK) and c-Jun N-terminal kinase (JNK) that can sequentially activate AP-1 complex composing of c-FOS and c-JUN [29]. AP-1 complex is a major transcription factor of *MMP-1* gene. Therefore, we hypothesized that the repression of *MMP-1* by PER3 might be under cAMP-mediated ERK and JNK signaling. To test this hypothesis, we examined expression levels of *c-FOS* and *c-JUN* after knockdown of PER3. Results showed that knockdown of PER3 did not influence the expression of *c-FOS* or *c-JUN* (Figure 2D,E), this suggests that PER3-mediated suppression of MMP-1 by cAMP is independent of ERK/JNK signaling.

### 2.3. LCE Increases PER Activity in HaCaT Cells

**Figure 3:**
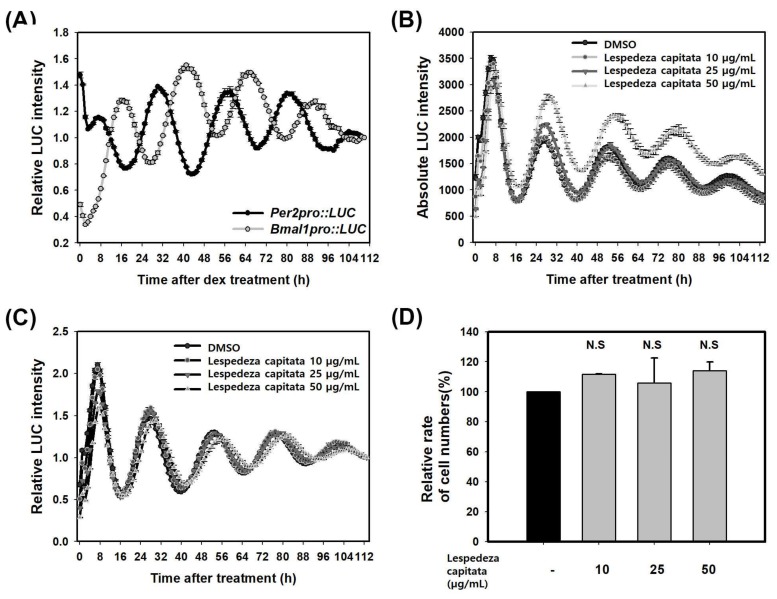
LCE increases *PER* promoter activity in HaCaT cells. (**A**) *Per2pro::LUC* and *Bmal1pro::LUC* reporter cell line. Circadian rhythms of single reporter cell clones were treated with 1 µM dexamethasone for 1 hour and bioluminescence was recorded for five days. Data were the de-trended time series of a representative single reporter cell clone. (**B**) Absolute bioluminescence of *Per2pro::LUC* treated with LCE for 24 h; (**C**) Relative bioluminescence of *Per2pro::LUC* treated with LCE for 24 h; (**D**) Relative rate of numbers in cells treated with LCE at 30 h after treatment was shown. Cells were counted using a cell counter. Data are presented as mean ± SEM from three replicated measurements (two-tail unpaired, *n* = 3, N.S.: not significant compared with DMSO (-)).

Our results obtained above suggest that skin circadian clock might be a new mechanism for regulating MMP-1. To confirm the role of circadian clock on regulating MMP-1 expression, we first searched for a modulator of circadian rhythm. To monitor skin circadian rhythm, we measured activities of *BMAL1* and *PER2* genes. It has been reported that *PER2* gene oscillates with high amplitude rather than *PER3* gene in fibroblasts and keratinocytes [15]. We generated *Per2pro-LUC*/*Bmal1pro-LUC* cell line containing firefly luciferase gene (LUC) under the control of a 526bp *PER2* promoter fragment from mouse genome or a 1.34-Kb *BMAL1* promoter fragment from human genome. These reporter constructs were stably integrated into HaCaT keratinocytes and single reporter clones were obtained. It has been reported that *PER2* promoter regions show high homology between mice and humans [37]. Circadian rhythms were synchronized with 1 µM dexamethasone for one hour and rhythmic luciferase activity of live cells was monitored for five days with a real-time monitoring system. Results showed that *PER2* or *BMAL1* promoter activity oscillated persistently for five days (Figure 3A). Reporter clones of *PER2* promoter activity with a period of 25.48 ± 0.17 h, a RAE of 0.12 ± 0.00, and a phase of 7.25 ± 0.25 was obtained. Those of *BMAL1* promoter activity with a period of 25.36 ± 0.07 h, a RAE of 0.13 ± 0.01, and a phase of 16.50 ± 0.29 (SEM, *n* = 3) were obtained (Table 1). Each rhythm expressed in an anti-phasic manner. These results revealed that skin circadian rhythm could be measured at cellular level in HaCaT cells using *Per2pro-LUC*/*Bmal1pro-LUC* cell line. We then searched for an activator of *PER2* activity using *Per2pro-LUC* reporter cell line and various plant extracts. We found several putative activators of *PER*. Among those, *L. capitata* extract (LCE) showed significant activation of *Per2pro-LUC* in a dose-dependent manner. After treatment with 50 µg/mL LCE, *PER2* activity was increased by 47% at 30 h after treatment compared with to that in untreated sample at 28 h after treatment (Figure 3B). LCE slightly lengthened the period of *PER2* promoter activity without changing RAE (Figure 3C, Table 1). These results showed *PER2* promoter activity of the control group had a period of 24.87 ± 0.02 h and a RAE of 0.16 ± 0.00 (SEM, *n* = 3) while that of the experimental group treated with 50 µg/mL LCE had a period of 25.18 ± 0.00 h and a RAE of 0.17 ± 0.00 (SEM, *n* = 3) (Table 1). Next, we determined whether the increase of *PER2* activity was caused by an increase in the number of cells or an increase of *PER2* activity in single cells. The number of cells incubated with LCE at 30 h after treatment when *PER2* expression was the highest was counted. The number of cells treated with LCE was not changed compared to that of untreated cells (Figure 3D). These results showed that LCE extract could increase *PER2* activity without affecting cell proliferation or cytotoxicity.

### 2.4. LCE Suppresses the Expression of MMP-1 through PER in HaCaT Cell

**Figure 4:**
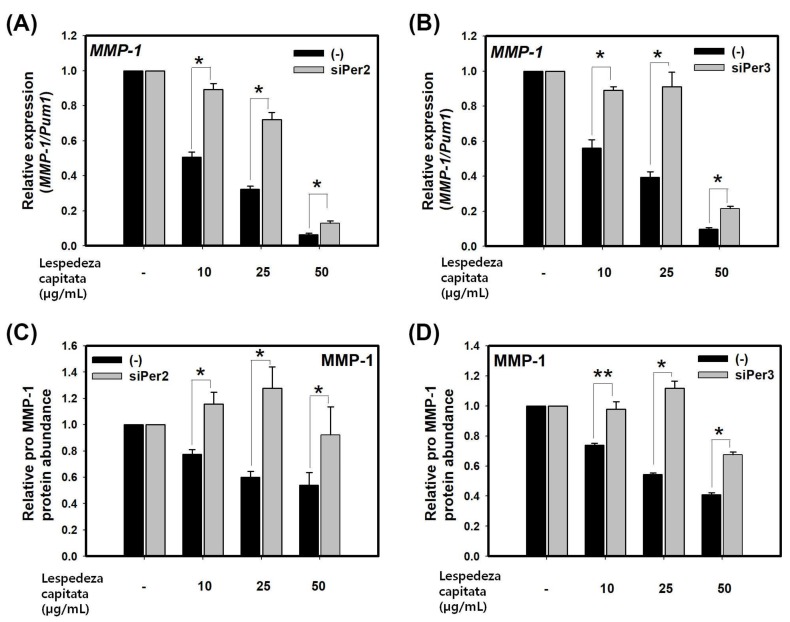
LCE suppresses the expression of MMP-1 through PER in HaCaT cells. Levels of MMP-1 transcripts (**A**) and pro MMP-1 protein (**C**) in HaCaT cells treated with control-siRNA and siPer2 for 48 h followed by treatment with LCE for 24 h. Levels of MMP-1 transcripts (**B**) and pro MMP-1 protein (**D**) in HaCaT cells treated with control-siRNA and siPer3 for 48 h followed by treatment with LCE for 24 h. Data are presented as mean ± SEM from three replicate measurements. Data is the relative value compared with respective control group (control-siRNA or siPer3 at DMSO) (one-way ANOVA, followed by Dunnett T3 post hoc, *n* = 3, * *p* < 0.05, ** *p* < 0.01 compared with control-siRNA at indicated concentration).

To determine LCE, PER activator, exerts inhibitory effect on expression of *MMP-1*, we measured expression levels of MMP-1 in LCE treated group using real-time PCR and ELISA. LCE significantly decreased MMP-1 at mRNA and secreted protein levels in a dose-dependent manner (Figure 4A–D). We then determined whether the inhibition of MMP-1 expression by LCE was mediated by PER. HaCaT cells were transfected with siPER2 and siPER3 for 48 h followed by treatment with LCE for 24 h. Silencing of PER2 or PER3 by siRNA partially alleviated the effect of LCE on the suppression of *MMP-1* at mRNA and secreted protein levels compared to treatment by control siRNA (Figure 4A–D). Taken together, these results indicate that LCE can suppress *MMP-1* expression through circadian clock gene PER. Therefore, activator of circadian clock might be a new mechanism to regulate MMP-1.

## 3. Discussion

The circadian clock imparts 24-h rhythmicity on gene expression and cellular physiology in the cell. It has been reported that disruption of circadian clock genes can lead to diverse defects on gene expression important for skin function. *Bmal1*-deficient mutant mice have shown upregulated ROS levels and reduced lifespans and various symptoms of premature aging, suggesting that it might play a role in aging by regulating genes involved in anti-oxidant process [38]. In human keratinocytes, CLOCK regulates the expression of Aquaporin (AQO)-3 and tissue inhibitor of metalloproteinase (TIMP)-3 that control hydration and photoaging, respectively [39,40]. Knockdown of PER has shown diverse defects in cell differentiation, wound healing, autophagy in aging, and tumor development [15,41,42,43,44]. In epidermal stem cells, increase in PER1 and PER2 confers a more deterministic propensity toward differentiation due to increased expression of differentiation markers IVL and loricrin [17]. In fibroblasts, PER protein can bind to RNA binding protein NONO and activate p160-Ink4A cell cycle check-point gene in circadian-dependent manner. Due to the role of PER on the cell cycle, loss of PER has shown defects in the wound healing process by reducing epidermal regeneration, dermal organization, and collagen deposition [41]. It is well-known that MMP also plays a role in would healing by degrading collagen and other ECM proteins comprising connective tissues. Our data showed that knockdown of PER increased MMP-1 expression. We hypothesize that changes in dermal organization and collagen deposition not only could be caused by activation of p160-Ink4A, but also by inhibition of MMP-1 by PER protein. In tumor development, PER1 suppresses expression levels of tumor-related genes including *c-MYC* gene [43]. Especially, activation of RAS/MAPK signaling increases the expression of MMP-1 but represses PER expression in tumor cell lines [45]. This is consistent with the fact that PER3 protein is a tumor suppressor gene [46]. These reports suggest that repression of MMP-1 by PER might be one mechanism involved in tumor development. Taken together, these results demonstrate that disruption of core clock genes affects gene expression and cellular processes. Therefore, reinforcing the level of core clock genes is necessary to sustain biological fitness.

The skin peripheral clock can be disrupted by external and internal signals such as UV radiation, pathogen, and aging [46,47,48]. Synthetic drug-like molecules and plant extracts that directly modulate the activity of key clock proteins might have potential to directly regulate clock-controlled genes important for skin function [49,50,51,52]. For pharmacological targeting of the skin circadian clock, many studies have established a viable model system using luciferase-based reporter system containing *BAML1* or *PER2* promoter fragment. In HaCaT keratinocytes, the circadian clock robustly oscillates in individual cells [53]. Therefore, we used this reporter system in HaCaT keratinocytes to study circadian rhythm in epidermal keratinocytes and screened for a modulator of circadian rhythm. The period, amplitude, phase, and level of circadian rhythm can be affected by certain molecules in a variety of manners. Such effects can be dependent on chronic or transient exposure to the molecule.

Our data showed that forskolin repressed the expression of MMP-1 while knockdown of PER3 partially restored its effect. A previous report has shown that cAMP signaling can repress MMP-1 expression through inhibiting ERK, JNK, and sequential AP-1 complex composed of c-FOS and c-JUN [29]. However, our results showed that knockdown of PER3 did not influence the expression of *c-FOS* or *c-JUN*. This means that another mechanism might be responsible for cAMP-mediated repression of MMP-1. It has been reported that activated PKA by cAMP can lead to activation of PER gene through CREB/CRE signaling [31,32,33]. Taken together, these results suggest that cAMP can repress the expression of MMP-1 not only through PKA-mediated inhibition of AP-1 complex, but also through PKA-mediated activation of PER.

The circadian clock is an endogenous timing mechanism that allows the anticipation of regular daily changes and the operation of biological processes at proper time in a day, thereby increasing fitness. In human epidermal stem cells, the intricate mechanism of the circadian clock keeps homeostasis by offering epidermal stem cells with environmental cues. During the day time, human keratinocytes induce DNA replication, protection to UV, and, subsequently, cell division. They offer protection against UV when they tend to duplicate their DNAs (more sensitive to UV-induced DNA damage). Our data showed that a morning component PER gene repressed the expression of MMP-1. *PER* gene peaks between late-night and morning transition. At 5 h after PER3, the genes involved in proliferation and protection against UV subsequently sequentially peak in human keratinocytes [17]. Thus, repression of MMP-1 by PER might be one of protection mechanisms against UV. Disruption of skin circadian clock by UV radiation and aging might contribute to skin aging and skin cancer. This is consistent with previous reports showing that PER protein plays a role in skin aging and skin cancer [42,43,45].

Our study is the first demonstration that Bmal1, Per1, Per2, and Per3 knockdown in HaCaT cells regulates expression of MMP-1. Especially, Per3 knockdown considerably upregulates MMP-1 expression. Also, the repression of MMP-1 by PER3 is mediated through cAMP signaling. It has been reported that MMP-1 is regulated by diverse signaling pathway such as MAPK signaling. We suggest the circadian clock can be a new mechanism for regulating MMP-1 expression in HaCaT cells. Based on our new findings, we anticipate investigating a role of PER gene in skin aging and skin cancer in vivo. 

## 4. Materials and Methods

### 4.1. Small-Interfering RNA Experiments and Pharmacological Treatment 

All siRNAs targeting the sequence of *BMAL1*, *PER1*, *PER2*, and *PER3* were purchased from Santacruz Biotechnology (sc-38165, sc-38171, sc-36209, sc-38173, Dallas, TX, USA). To detect off-target effects, a non-targeting 20–25 nt siRNA (sc-37007) was used as a negative control siRNA. For transfection, HaCaT cells were seeded to white 12-well cell culture plates at density of 1.1 × 105 cells/well. After 24 h of culture, cells were transfected with 12.5 nM of siRNA using Lipofectamine 2000 (Invitrogen, Carlsbad, CA, USA) and Opti-MEM (31985062, Gibco, Waltham, MA, USA) and according to manufacturers’ instructions. At 72 h after transfection, cells were harvested to analyze mRNA expression.

For forskolin and H-89 treatment, HaCaT cells were seeded to white 12-well cell culture plates at density of 1.1 × 105 cells/well. Cells were kept in DMEM containing 1% FBS. After 48 h, cells were treated with 1 and 10 µM FSK or 10 µM H-89 purchased from Cell signaling technology (#3828, #9844, Danvers, MA, USA). After 24 h of treatment, cells were harvested for analyzing mRNA expression. For FSK and siRNA co-treatment, HaCaT cells were seeded to white 12-well plates (1.1 × 105 cells/well). After 24 h, cells were transfected with 12.5 nM of siRNA using Lipofectamine 2000 (Invitrogen, Carlsbad, CA, USA) and Opti-MEM according to manufacturer’s instructions. At 48 h after transfection, cells were washed with PBS, treated with 1 or 10 µM forskolin, and transfected with siPer3 or control siRNA for 24 h. Cells were then harvested to analyze transcripts and protein levels. 

For *Lespedeza capitata* extract (LCE) and siRNA treatment, HaCaT cells were seeded to white 12-well cell culture plates at density of 1.1 × 105 cells/well. After 24 h of incubation, cells were transfected with 12.5 nM of siRNA using Lipofectamine 2000 (Invitrogen, Carlsbad, CA, USA) and Opti-MEM (31985062, Gibco, Waltham, MA, USA) according to manufactures’ instructions. At 48 h after transfection, cells were washed with PBS and treated with LCE (10, 25, and 50 μg/mL) followed by transfection with siPer2/siPer3 or control siRNA for 24 h. Cells were then harvested for analyzing transcripts and protein levels.

### 4.2. RNA Isolation and Quantitative Real-Time RT–PCR

We independently prepared samples for assays in triplicates. Total RNA was extracted using Qiagen RNeasy Mini Kit (Qiagen, Hilden, Germany) following the manufacturer’s instructions. Complementary DNA was obtained by reverse transcription of 1 μg of total mRNA using amfiRivert cDNA synthesis platinum master mix (GenDEPOT, Katy, TX, USA) following the manufacturer’s instructions. Real-time reverse transcription PCR was done using an ABI PRISM 7300 (Applied Biosystems, Foster City, CA, USA). For a 20 μL reaction, cDNA template was mixed with primers (final concentration of 300 nM) and 10 μL of SYBR Green PCR Master Mix (4309155, Applied Biosystems, Foster City, CA, USA). PCR was performed with 40 cycles of 95 °C for 15 s, 55 °C for 40 s, and 72 °C for 30 s after an initial incubation at 95 °C for 10 min. Primer sequences are listed in Table 2. Each value was normalized with that of RNA Binding Family Member (*PUM1*) and set by the maximum value of control group.

### 4.3. MMP1 Protein Determination by ELISA

Pro MMP-1 protein levels in cell culture supernatants were quantified by ELISA (Human Pro MMP-1; DMP100; R&D Systems, Minneapolis, MN, USA) according to the manufacturer’s instructions.

### 4.4. Establishment of a Stable Cell Line for Per2/Bmal1 Promoter-Based Reporter Gene Assay

Each plasmid pGL4.15 expressing a firefly luciferase (LUC) purchased from Promega (# 9PIE670, Madison, WI, USA) under the control of a 526bp *PER2* promoter fragment from mouse genome and plasmid pGL4.27 expressing LUC purchased from Promega (# 9PIE845, Madison, WI, USA) under the control of a 1.34-Kb *BMAL1* promoter fragment from human genome was stably integrated into HaCaT keratinocytes. After hygromycin selection (0.2 μg/mL; Sigma, Ronkonkoma, NY, USA), one colony was cultured into 24-well cell culture plates and sub-cultured sequentially into 12-well and 6-well plates for five weeks for hygromycin selection. After hygromycin selection, single-cell-derived sub-clones were analyzed in bioluminescence monitoring over five days following treatment with 1 µM dexamethasone for synchronization. Consequently, *Per2pro-LUC*/*Bmal1pro-LUC* stable cell line expressing *PER2*/*BMAL1* promoter and firefly luciferase gene was obtained.

### 4.5. Bioluminescence Recording

For live-cell bioluminescence monitoring, HaCaT reporter cell line was seeded to white 96-well plates (655083, Griner Bio One, Monroe, CA, USA) at density of 1.4 × 104 cells/well. After two days, cells were synchronized with 1 µM dexamethasone (D1756, Sigma Aldrich, St. Louis, MO, USA) for one hour. The medium was then changed to a recording media: DMEM containing 1% fetal calf serum, 1% penicillin/streptomycin, 10 mM HEPES, pH 7.4, 0.2 mg/mL Hygromycin, and 1 mM luciferin. Bioluminescence recordings were taken every hour for five days using Infinite M200 (Tecan, Männedorf, Switzerland). Raw data were de-trended by dividing data points by 24-h running average. Relative luminescence intensities from data were imported into Biological Rhythms Analysis Software System (BRASS, provided by Andrew Millar lab). Periods and relative amplitude error (RAE) were analyzed using FFT-NLLS suite.

### 4.6. Extraction of L. capitata

*Lespedeza capitata* was purchased from Jeju Island, South Korea. The raw extract desiccated by using hot air drier at 50 °C overnight was grinded. Dried and pulverized aerial parts of *L. capitata* (1 kg) were extracted with 70% ethanol (20 kg) at 80 °C for 3 h. *L. capitata* extract (LCE) was filtered through 5-μm charcoal filter and concentrated by evaporation. Concentrate was dried using a freeze dryer and powdered

### 4.7. Pharmacological Treatment for Bioluminescence Recording

To determine the effect of LCE on skin circadian rhythm, *Per2pro-LUC* cells were seeded into white 96-well plates (655083, Griner Bio One, Monroe, CA, USA) at density of 1.4 × 104 cells/well. After two days, cells were washed with PBS and treated with LCE (10, 25, and 50 µg/mL) for 24 h. Cells were then washed with PBS and replaced with freshly made recording medium. Bioluminescence was taken every hour for five days.

### 4.8. Determination of Cell Number

To determine cell number, cells were washed with PBS and dissociated by trypsin. Cells were then resuspended with DMEM containing 10% FBS and cell number was measured using a cell counter (Millipore, Billerica, MA, USA). Cell number counted by a Scepter cell counter was based on Coulter principle of impedance-based particle detection [54]. Cells ranging in diameter between 10 to 22 μm were counted as surviving cells [55].

### 4.9. Statistical Analysis

Data are presented as mean ± SEM from three replicate measurements. Continuous variables were tested for normal distribution by using the Shapiro–Wilk test and for homogeneity of variance test by using Levene test. All data showed normal distribution. Two-tail unpaired *t*-test and one-way ANOVA followed by Bonferroni or Dunnett T3 post hoc depending on homogeneity of variance were used as appropriate for statistical differences. All calculations were carried out by using SPSS statistics.

## Figures and Tables

**Table 1:** Analysis of circadian rhythm. Relative luminescence intensities from each data in Figure 3A, C imported into the Biological Rhythms Analysis Software System (BRASS), and periods and relative amplitude error (RAE) were analyzed using the FFT-NLLS suite.

		***Per2pro::LUC***	***Bmal1pro::LUC***		
Period (h)	Average	25.48	25.36		
	S.E	0.17	0.07		
Amplitude	Average	0.24	0.26		
	S.E	0.01	0.00		
R.A.E	Average	0.12	0.13		
	S.E	0.00	0.01		
Phase (h)	Average	7.25	16.50		
	S.E	0.25	0.29		
		**DMSO**	***Lespedeza Capitata***		
			**10 µg/mL**	**25 µg/mL**	**50 µg/mL**
Period (h)	Average	24.87	24.72	25.01	25.53
	S.E	0.02	0.03	0.01	0.04
Amplitude	Average	0.29	0.28	0.27	0.22
	S.E	0.00	0.00	0.00	0.00
R.A.E.	Average	0.16	0.17	0.17	0.19
	S.E	0.00	0.00	0.00	0.00
Phase (h)	Average	7	7	7	8
	S.E	0	0	0	0

**Table 2:** List of primer sequence

Gene Name	Accession Number	Primer Sequence	
*PUM1*	NM_014676	hPum1-F	5′-CGGTCGTCCTGAGGATAAAA-3′
		hPum1-R	5′-CGTACGTGAGGCGTGAGTAA-3′
*BMAL1*	NM_001668	hBmal1-F	5′-TTAAGAGGTGCCACCAATCC-3′
		hBmal1-R	5′-CTTCCCTCGGTCACATCCTA-3′
*PER1*	NM_002616	hPer1-F	5′-GGACACTCCTGCGACCAG-3′
		hPer1-R	5′-GGGAGTGAGGTGGAAGATCTAA-3′
*PER2*	NM_022817.2	hPer2-F	5′-TTCCCAGCAAACGTCCCAG-3′
		hPer2-R	5′-GGTGCGTACCTACTCCCGT-3′
*PER3*	NM_016831	hPer3-F	5′-GCGCATTCTCATGACATACC-3′
		hPer3-R	5′-TGCTGCTGCCTCATACTTTC-3′
*c-FOS*	NM_005252.3	hc-Fos-F	5′-CTACCACTCACCCGCAGACT-3′
		hc-Fos-R	5′-AGGTCCGTGCAGAAGTCCT-3′
*c-JUN*	NM_002228.3	hc-Jun-F	5′-CCAAAGGATAGTGCGATGTTT-3′
		hc-Jun-R	5′-CTGTCCCTCTCCACTGCAAC-3′
*MMP-1*	NM_002421.3	Qiagen (PPH00120B)	
